# Trampoline Park Injuries and Their Burden on Local Orthopaedic and Emergency Services

**DOI:** 10.29252/beat-070212

**Published:** 2019-04

**Authors:** Stevan J. Jordan, Christopher J. To, Roozbeh Shafafy, Amelia E. Davidson, Kathryn Gill, Matthew C. Solan

**Affiliations:** 1 *Trauma and Orthopaedic Department, Royal Surrey County Hospital NHS Trust, Guildford, GU2 7XX, UK*; 2 *Trauma and Orthopaedic Department, Brighton and Sussex University Hospitals NHS Trust, Brighton, BN2 5BE, UK*

**Keywords:** Trampoline park injuries, Local, Orthopaedic; Emergency, Services

## Abstract

**Objective:**

To investigate the incidence of trampoline park injuries (TPIs) at a local recreational facility and to quantify the burden on emergency and orthopaedic services at our institute.

**Methods:**

All patients that presented to the Emergency Department (ED) from the trampoline park via ambulance from July 2014 to November 2015 were included in the study. Patients’ medical records were reviewed for clinical details including date, location and type of injury, treatment received, length of stay and outpatient follow-up. A cost analysis was performed to estimate the financial impact of each injury.

**Results:**

A total of 71 patients were included in the study, with a mean age of 20 (7-48). Soft tissue sprains (n=29, 41%) and fractures (n=25, 35%) were the most common injuries, with the majority occurring in the lower limb. Two patients sustained open tibial fractures necessitating transfer to level 1 trauma centres. Fourteen patients (20%) underwent surgery, predominantly requiring open reduction and internal fixation. Overall, 18 patients (25%) required admission to hospital with mean length of stay of 2 days. The cost for pre-hospital, emergency and in-patient care amounted to over £80,000.

**Conclusion:**

TPIs pose a significant financial cost for local orthopaedic and emergency services. Contrary to studies evaluating home trampoline injuries, the majority of fractures at trampoline parks occurred in the lower limbs. Improved injury prevention strategies are required to help reduce morbidity and lower the financial implications for local NHS trusts.

## Introduction

Trampolining is a popular recreational activity.  Its debut appearance in the Sydney year 2000 Games of the XXVII Olympiad has further propelled its widespread affection as a sport.  This is reflected by the large increase in trampoline sales over the last 15 years in the United Kingdom [1]. More recently, several indoor recreational trampoline parks have emerged throughout the UK to capitalise on this growing popularity. A similar trend has been seen in the United States of America (USA) [2].

Trampoline-related injuries are common in both the paediatric [3, 4] and adult populations.[5] Injuries commonly occur following falls on the mat, falls off the trampoline apparatus, impact with trampoline frames or springs, and in collisions with simultaneous users [6-8]. Although the most common trampoline injuries described are simple soft tissue sprains and contusions, serious injuries including cervical spine fractures have been reported [9-11]. In light of the risks associated with trampoline use, the American Academy of Pediatrics (AAP) and the American Academy of Orthopedic Surgeons (AAOS) have published several policy statements consistently discouraging the recreational use of trampolines [12-15]. The most recent statement by the AAP also recognises the specific lack of data regarding the safety of recreational trampoline parks and recommends strict adherence to its guidelines for trampoline use at such venues [14].

**Fig. 1:**
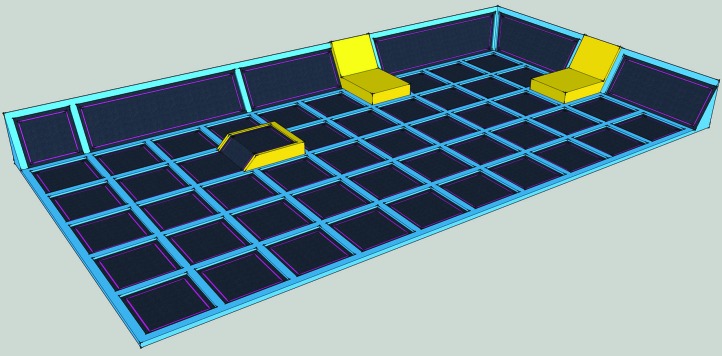
Schematic of a typical trampoline park design

Indoor recreational trampoline parks typically consist of multiple adjacent trampoline mats with padded borders (Figure 1). The boundaries of the design usually include steeply inclined trampoline mats or padded walls that prevent users falling off the trampolines. These parks regularly host large groups of participants and have multiple users at any one time. Safety guidelines vary across parks, and whilst multiple users are often prohibited from jumping on the same mat, individual somersaults are often permitted [16, 17].

Although trampoline related injuries are well studied in the domestic home environment, there is a paucity of data concerning recreational trampoline park injuries (TPIs). The aims of this study were to report the number, anatomical location and type of TPI, along with their associated costs, of all patients that presented from a local trampoline park to the emergency department (ED) of our institute, a district general hospital, from July 2014–November 2015. 

## Materials and Methods

 *Study population*

  All patients attending the ED of our institute, the Royal Surrey County Hospital (a district general hospital in the South of England), via ambulance with injuries sustained at the local recreational trampoline park were included in the study. Data was collected retrospectively over a sixteen-month period between 1st July 2014 and 1st November 2015. Attendees were identified through the South East Coast Ambulance Service (SECAmb) database of emergency call-outs using the name, address and postcode of the local trampoline park. 

*Study protocol*

 The patients’ medical records were interrogated to confirm that all injuries sustained at the trampoline park were secondary to trampoline use. Clinical details of each case, including the date of injury, age, anatomical location and type of injury, treatment received, length of stay and outpatient follow-up were recorded. Financial data regarding the cost of patients’ hospital treatment for emergency, inpatient and outpatient care was determined through the National Health Service’s (NHS) national tariff payment system, ‘Payment by Results’ (PbR) [18]. PbR is the payment system in England under which commissioners pay NHS healthcare providers for each patient seen or treated. The amount paid is determined by the complexity of the patient's healthcare needs. Nationally derived codes correspond to specific tariffs i.e. there is a set code, and therefore tariff, for the treatment of femoral fractures. Codes include healthcare resource group (HRG) codes for ED attendances; HRG, diagnosis (ICD-10) and intervention (OPCS-4) codes for in-patient treatment; and treatment function codes (TFC) for outpatient appointments.

## Results

**Fig. 2:**
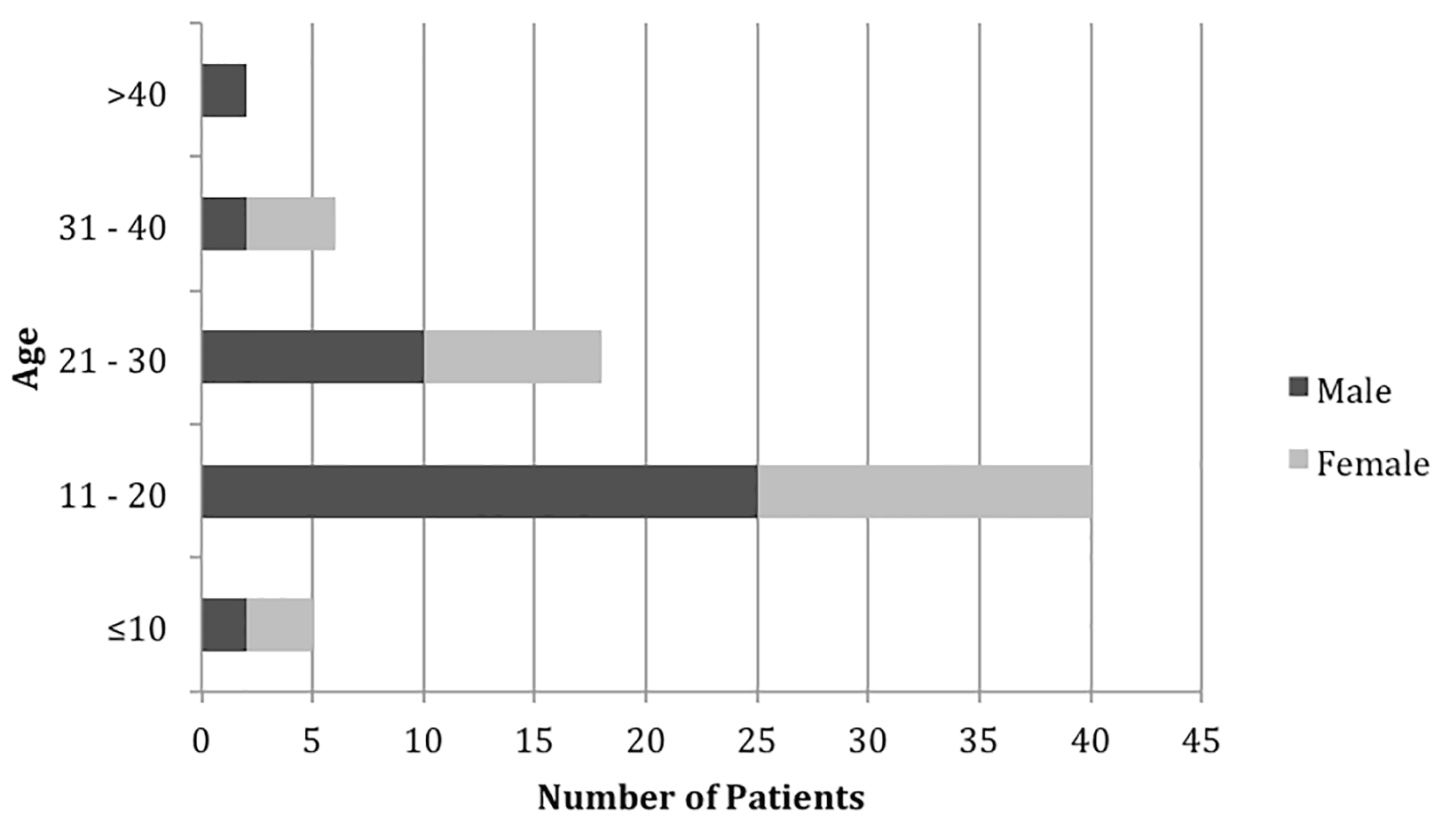
Distribution of injuries by age and gender

**Fig. 3:**
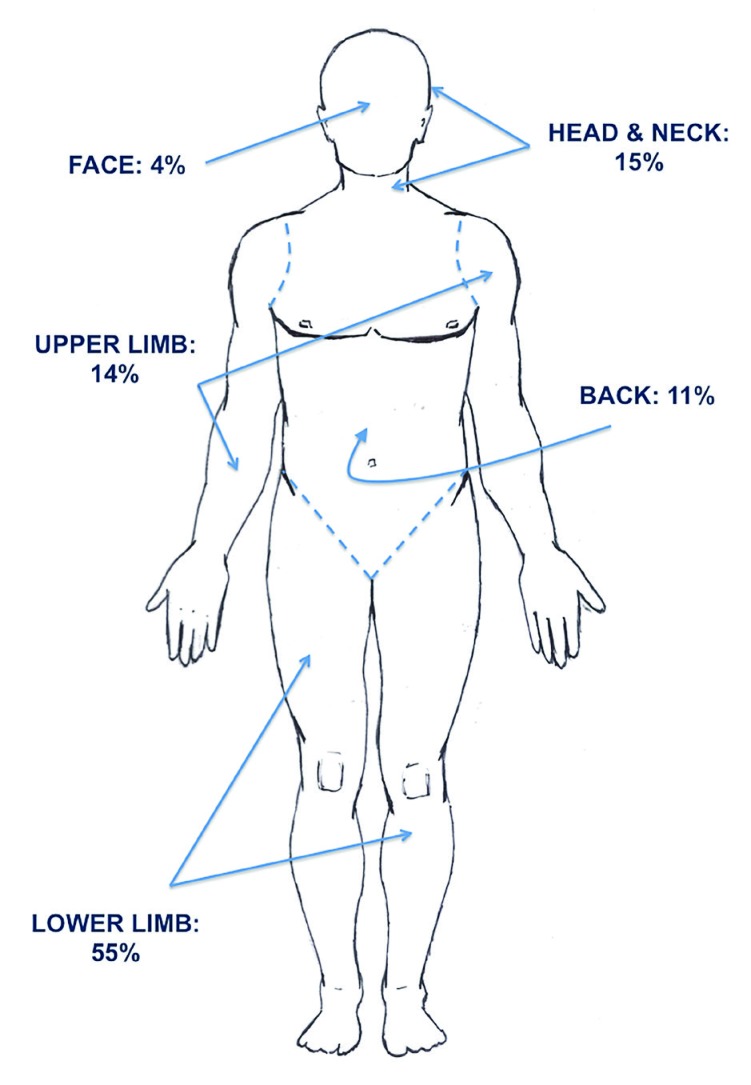
Distribution of injury by anatomical location

**Fig. 4:**
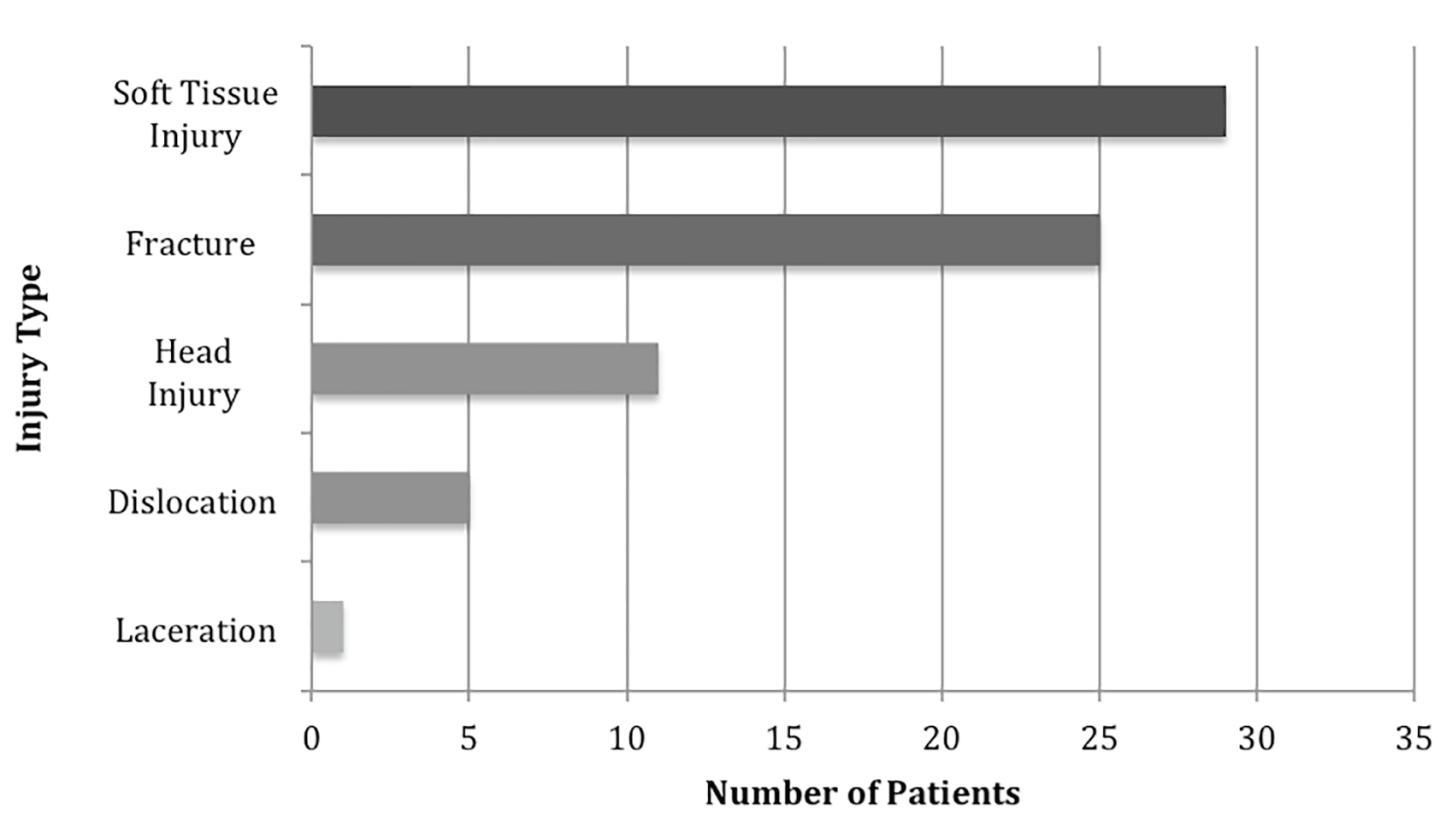
Distribution of injury type

Over the sixteen-month study period, a total of 71 patients were brought to ED by ambulance with injuries sustained at the local recreational trampoline park. Of those, 41 were male and 30 females, with a mean age of 20 years (range 7-48 years; Figure 2). There were 26 paediatric patients (age <16 years). The distribution of injuries appeared to be largely similar between paediatric and adult patients with the majority of injuries occurring in the lower limbs for both fractures (Table 1) and soft tissue injuries (Table 2) (Figure 3). Differences between the two populations include the number of joint dislocations (5 in adults vs. 1 in children) (Table 2). The paediatric population also sustained fractures specific to paediatric morphology such as supracondylar fractures and physeal injuries.  Soft tissue sprains were the most common diagnosis, followed by fractures, head and neck injuries, joint dislocations and lacerations (Figure 4). 

**Table 1:** Incidence of fractures by anatomical location

		**Number of patients**		
**Fracture Type**		**Paediatric**	**Adult**	**Total**
**Lower Limb**	Ankle	2	5	7 (28%)
	Tibia/Fibula – Shaft	1	4	5 (20%)
	Tibia- Proximal Physis	1	0	1 (4%)
	Tibial Plateau	0	1	1 (4%)
	Femur- Distal Physis	1	0	1 (4%)
	Talus	0	1	1 (4%)
	Total (Lower Limb)	5	11	16 (64%)
**Upper Limb**	Radius/Ulna	1	1	2 (8%)
	Supracondylar	1	0	1 (4%)
	Epicondylar	0	2	2 (8%)
	Total (Upper Limb)	2	3	5 (20%)
**Spine**	Lumbar Vertebra	0	2	2 (8%)
**Facial**	Nasal	0	2	2 (8%)
**Total Fractures**		7	18	25 (100%)

**Table 2:** Incidence of soft tissue injuries by anatomical location

		**Number of patients**		
**Location**		**Paediatric**	**Adult**	**Total**
**Lower Limb**	Ankle sprain	6	10	16 (33%)
	Knee sprain	0	6	6 (12.5%)
	Patella dislocation	1	0	1 (2.1%)
	Total Lower limb	7	16	23 (47.9%)
**Head**		7	4	11 (22.9%)
**Neck**	Sprain	2	0	2 (4.2%)
**Upper Limb**	Elbow dislocation	1	1	2 (4.2%)
	Shoulder dislocation	0	4	4 (8.3%
**Back**	Sprain	3	3	6 (12.5%)
**Totals**		20	28	48 (100%)

**Fig. 5:**
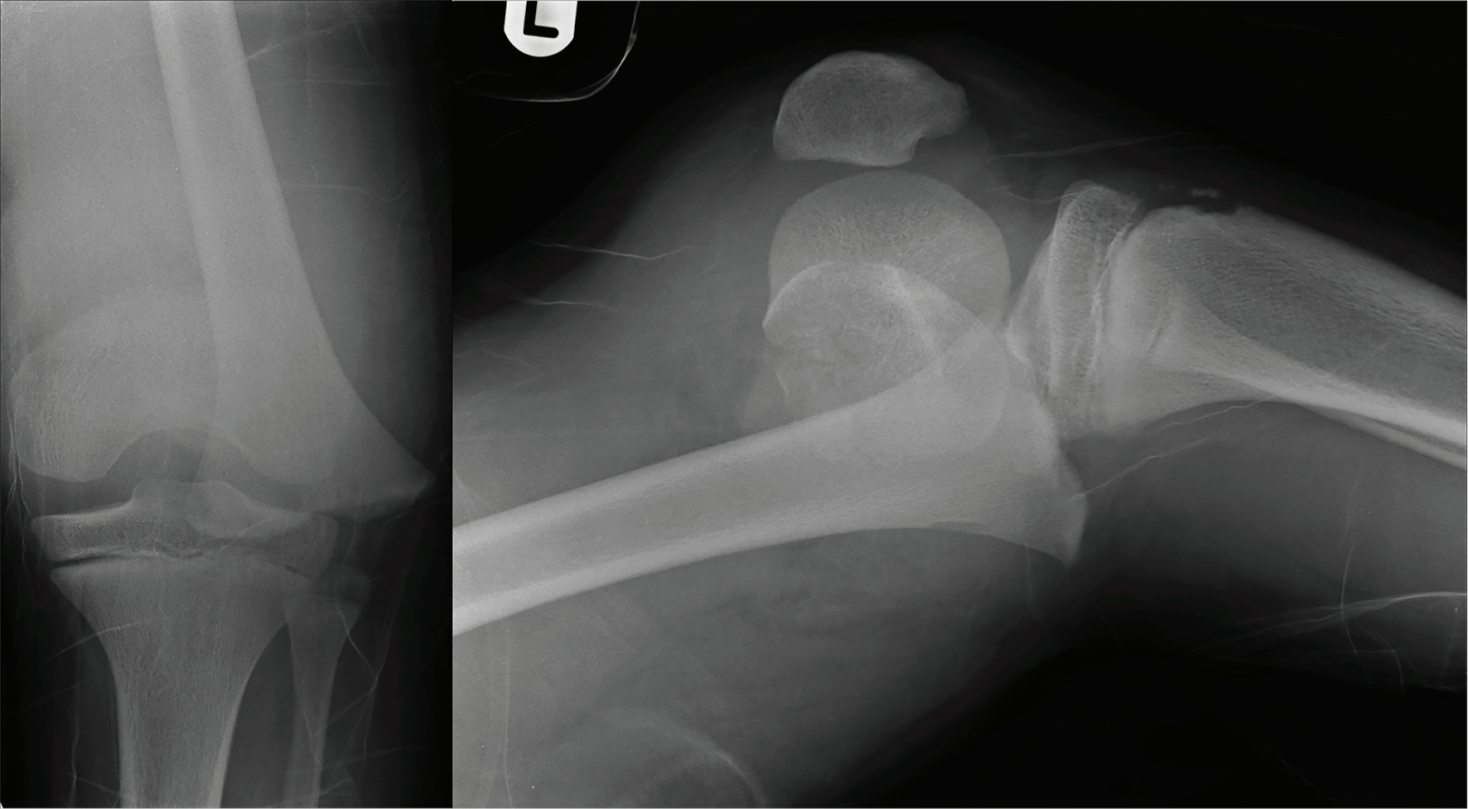
Plain antero-posterior and lateral radiographs demonstrating a fracture through the distal femoral physis with complete anterior displacement

Of the patients in this study, 14 (20%) required operative intervention. Twelve patients were treated with open reduction and internal fixation, whilst the remaining two underwent manipulation under anaesthesia and insertion of percutaneous Kirschner-wires (K-wires). Two of the patients required an additional visit to theatre. The most clinically significant injuries were: a completely displaced fracture of the distal femoral physis (Figure 5) requiring closed reduction and fixation with percutaneous K-wires; and two open tibial fractures (Gustillo grade II) requiring urgent transfer to the local level one trauma centre for definitive surgical care with plastic surgery involvement. Head and neck injuries were sustained by 11 patients (15%), of which all were soft tissue muscle sprains.  None of these had any neurological deficits. Overall, a total of 18 patients (25%) required admission for on-going care. Of these, 7 were paediatric patients and 11 adults (aged over 16). The mean length of stay was 2 days (range 1-7), excluding the two patients transferred to the local level one trauma centre. A total of 38 outpatient appointments were made for 12 patients at fracture clinic, with a further 11 non-resident patients being followed up at their local respective hospitals. A total of 13 physiotherapy referrals were made locally. The trampoline park receives an estimated 300,000 jumpers annually [19] meaning in an average month of 25,000 visitors, 4.4 people required a transfer to the local ED via ambulance per month.  This equates to an estimated frequency of about 1 patient every 7 days.

 *Cost analysis*

 The total estimated cost of TPI encountered at our hospital, calculated using the NHS’s national tariff PbR payment system [18] and taking into account emergency care, hospital admission, inpatient care, surgical intervention, outpatient physiotherapy and fracture clinic follow-up amounted to £64,388 (Table 3).   The total cost for the South East Coast Ambulance  was over £16,500, calculated using the National Audit Office’s average call-out cost per incident for SECAmb [20]. Twenty-six paediatric patients sustained TPIs with a mean healthcare cost of £905 (range £298 to £4,158) compared with 45 adult patients with a mean cost of £1,274 (range £298 to 4789).  The cost for pre-hospital, emergency and in-patient care in total amounted to over £80,888. 

**Table 3:** Costs associated with TPIs

	**Paediatric (n=26)**	**Adult** **(n=45)**	**Total**
**Ambulance Callout**	£6,042	£10,458	£16,500
**Emergency Department Care**	£2,490	£5,319	£7809
**Inpatient Care**	£13,875	£38,154	£52,029
**Outpatient Care**	£1,138	£3,412	£4,550
**Total Cost**	£23,545	£57,343	£80,888

## Discussion

To our knowledge, this is the first study to investigate trampoline park injuries and their financial impact on emergency healthcare provision in the UK. In domestic trampoline use, fractures and dislocations mostly occur when participants fall off the trampoline and predominantly affect the upper extremity [6, 21-23]. Injuries that occur on the mat, whilst more frequent [6, 8, 22], usually result in soft tissue sprains of the lower limb [6]. Our study demonstrated a comparable result. In contrast to domestic trampoline use however, fractures at the trampoline park were most prevalent in the lower limb, accounting for 35% of all TPIs. Several severe lower limb injuries were seen with two open tibial fractures and one off-ended distal femoral physeal fracture. Fractures, as in other studies, also represented the main injury type for younger patients (<16 years) [21, 24, 25], but were again more prevalent in the lower limb. These findings are supported by Kasmire *et al*. who report that TPIs result in a higher prevalence of lower extremity injuries when compared to domestic trampoline use [2]. 

 In this study, head and neck injuries were the second most common injury type by location, representing 15% of all TPIs. This is similar to other studies looking at domestic trampoline use that report between 10-17% of all trampoline injuries occurring in the head and neck region [6, 21, 23]. Isolated neck injuries were seen in 7% and were all stable soft tissue muscular sprains. None of the patients who sustained head and neck injuries had any neurological deficit. The mechanism of injury was not recorded for the majority of these patients, but two cases documented neck injuries were sustained while attempting somersaults. High-risk manoeuvres, including somersaults, are strongly discouraged by a large number of studies due to the risk of cervical spine injury [4, 7, 9, 11]. The AAP states clearly that somersaults and back-flips should not be performed in the recreational setting as a result of this risk [14]. Cervical spine injuries have also been documented to occur in trampoline parks [2]. Hospital admission was required in 25% of the TPIs presenting to our ED. A larger proportion than reported by Nysted *et al*., [6] The most common reason for admission was a fracture requiring surgical fixation or manipulation under anaesthesia. Follow up in fracture clinic was required for all patients who sustained fractures.

The estimated cost of all TPI encounters with the hospital, derived by the NHS’s national tariff payment system, amounted to £64,388; ED attendances accounted for £7,809, inpatient care, including surgery £52,029, and outpatient care £4,550. Although these figures do not appear vast, for a single recreational venue they pose a significant financial burden on an ever-tightening healthcare budget. The amount equates to the cost of employing an extra three foundation year doctors, or three band 6 nurses. Further work to establish better safety guidelines and reduce TPI risk would potentially reduce admissions and bring the associated healthcare costs down.

Long-term morbidity resulting from TPIs was not measured in this study, however it has been reported that these injuries can have long-term impact on physical function.  Patient recorded outcome measures revealed 33% of patients suffered from persistent pain at 6 months’ post injury. At the same timepoint, 32% of patients still had some form of persistent disability with 14% of patients who were previously employed were unable to return to work because of their injuries [4]. These figures may help to provide participants with an informed decision when partaking in trampolining activities and help facilitators engage in injury prevention measures.

 *Suggestions for prevention*

 Trampoline parks commonly have large groups of users of varying age and size on the mats in the park at any one time. The park rules often demand only one user per mat [16], but this fails to prevent multiple users from ending up on the same mat occasionally, either accidentally or intentionally. Many authors have considered multiple jumpers on the mat in domestic trampoline use as an important risk factor for trampolining injuries [6, 8, 21]. The mass of participants on the mat also influences injury rates [26], with smaller jumpers over 10 times more likely to sustain injuries than their heavier counterparts [26]. The energy transferred when users jump out of phase can be greater than landing on solid ground from a height of over 2.5m [27]. As well as employing park wardens to monitor the one user per mat policy, it may be beneficial to designate specific jumping areas for users of a certain age or weight. Further work to investigate TPIs resulting from multiple jumpers on the same mat would be valuable. 

The trampoline park in our study openly permitted somersaults as long as they weren’t performed in succession [16]. Given the inherent risk associated with somersaults specifically with regards to head and neck injuries as mentioned above, performing these manoeuvres in trampoline parks should also be prohibited.

Trampoline park injuries in the UK have not previously been investigated to this detail. Whilst the perception may be that recreational trampoline parks are safer due to the presence of trained staff and official equipment, this study has in fact revealed that serious life-changing injuries do occur at these parks and acknowledges that such installations pose significant costs for local healthcare services. In addition, the pattern of injuries sustained at these venues appears to differ to that of domestic trampoline use with a greater proportion of serious lower limb fractures.The main limitation to this study was potential loss of capture of all patients sustaining TPIs. All patients who either self-presented to ED not via ambulance, had injuries necessitating direct ambulance transfer to a major trauma centre (e.g the multiply injured patient), or were taken to an alternative local hospital would not have been included in the study. As a result, our findings may underestimate the true burden of TPIs. A second limitation was our inability to draw conclusions about the mechanism of injury whilst trampolining within the park, preventing us from making direct recommendations about park safety improvements or preventative measures.>However, whilst these injuries do occur, it is important to recognise the relative health benefits enjoyment that such installations offer in helping children and adults exercise and keep fit. It is therefore evident that further studies are required to evaluate the safety of trampoline parks to help establish improved safety guidelines. Collaborative efforts with trampoline parks would be beneficial to help identify the common mechanisms by which these injuries occur and provide data on injury rates for this activity to advise what appropriate preventative measures should be in place. This would also enable a risk comparison between trampoline parks and other popular activities to be investigated.

## Conflict of Interest:

None declared.
